# Stem Cell Basis for Fractal Patterns: Axillary Meristem Initiation

**DOI:** 10.3389/fpls.2021.805434

**Published:** 2021-12-17

**Authors:** Ying Wang

**Affiliations:** College of Life Sciences, University of Chinese Academy of Sciences, Beijing, China

**Keywords:** axillary meristem, stem cell, transcription factors, phytohormone, epigenetic modifications, boundary, mechanical stress, fractal patterns

## Abstract

Whereas stem cell lineages are of enormous importance in animal development, their roles in plant development have only been appreciated in recent years. Several specialized lineages of stem cells have been identified in plants, such as meristemoid mother cells and vascular cambium, as well as those located in the apical meristems. The initiation of axillary meristems (AMs) has recently gained intensive attention. AMs derive from existing stem cell lineages that exit from SAMs and define new growth axes. AMs are in fact additional rounds of SAMs, and display the same expression patterns and functions as the embryonic SAM, creating a fractal branching pattern. Their formation takes place in leaf-meristem boundaries and mainly comprises two key stages. The first stage is the maintenance of the meristematic cell lineage in an undifferentiated state. The second stage is the activation, proliferation, and re-specification to form new stem cell niches in AMs, which become the new postembryonic “fountain of youth” for organogenesis. Both stages are tightly regulated by spatially and temporally interwound signaling networks. In this mini-review, I will summarize the most up-to-date understanding of AM establishment and mainly focus on how the leaf axil meristematic cell lineage is actively maintained and further activated to become CLV3-expressed stem cells, which involves phytohormonal cascades, transcriptional regulations, epigenetic modifications, as well as mechanical signals.

## Introduction

Plants display incredible organogenetic ability and developmental plasticity throughout the whole life cycle, which comes from the sustained activity of stem cells in apical meristems. In addition, stem cells contained in apical meristems are protected from harsh conditions including drought, high salinity, high temperatures, and UV irradiation accompanying the movement from aquatic to terrestrial habitats. Therefore, these stem-cell harboring structures comprise the “fountain of youth” for indeterminate plant growth (Baurle and Laux, 2003) and represent an important innovation during plant colonization of land (Pfeiffer et al., 2017; Nishihama and Naramoto, 2020). Hence, specification of stem cells and establishment of stem-cell harboring structures is an important problem to study in the field of plant development.

Stem cells are formed not only during embryogenesis, but also in postembryonic development when axillary meristems (AMs) initiate. Stem cells are specified in the center of newly established AMs. AM formation involves cell fate determination, meristematic cell maintenance, and meristem organization. AMs form in the leaf axil located at the leaf base, giving rise to lateral branches as new growth axes and conferring seed plants a ramifying and fractal architecture. Because of the activity of AMs, the shoot is self-replicating and scalable at different levels of branching, generating a fractal geometry pattern. The reiterative AM initiation from boundary regions suggests that it is a highly robust and precisely controlled developmental process. The establishment of AMs combined with the subsequent outgrowth of AMs determines the number and activity of lateral branches and therefore significantly influences crop yield. Each AM has the same developmental potential as the shoot apical meristem (SAM) and is required for new lateral organ formation on lateral branches. Once the AM begins to express marker genes such as *WUSCHEL* (*WUS*) and *CLVATA3* (*CVL3*), we consider that new stem cells have emerged.

## A Leaf Axil Meristematic Cell Lineage

Given that cells in AMs originate from the SAM, the origin and establishment of the stem cell niches in AMs is of particular interest to the scientific community. AMs are formed at the base of their subtending leaves on the adaxial side, which faces toward the SAM. One possible scenario is that stem cells detach from the SAM and retain their fate during leaf formation. Conversely, because AMs are clonally related to their subtending leaves (Furner and Pumfrey, 1992; Irish and Sussex, 1992), it is also possible that AMs arise from differentiating tissues and the new stem cell niches are reconstituted *de novo* in AMs. Whether AMs originate as detached meristems or *de novo* induced ones had remained as a long-debating question in developmental biology (Steeves and Sussex, 1989; Long and Barton, 2000). Although these seem to be two incompatible models, recent studies with live-imaging on leaf axil cells suggest that in fact they can be reconciled and combined into a single two-step regulation model. *SHOOTMERISTEMLESS* (*STM*) encodes an important KNOTTED-like transcription factor required for both embryonic shoot apical meristem (SAM) formation and AM formation in leaf axils, suggesting that SAM and AM share a common molecular regulatory mechanism. AM formation can be divided into two steps, meristematic cell lineage maintenance and stem cell activation (Wang and Jiao, 2018). In the meristematic cell lineage maintenance phase, cells in the leaf axils express low levels of *STM* (Burian et al., 2016; Shi et al., 2016), which is not sufficient for AM initiation but necessary to keep stem cell competence. These cells lack the expression of either *CLV3* or *WUS*, and are distinct from stem cells in the SAM central zone. In the subsequent stem cell activation phase, *STM* expression is highly elevated beyond a certain threshold, which supports the induction of AM initiation and bulging. Multiple studies have shown that cells with low or no *STM* expression are no longer able to initiate AM formation (Shi et al., 2016; Cao et al., 2020). *WUS* and *CLV3* are subsequently expressed in the AM to reestablish stem cells (Wang et al., 2017). These findings suggest that there exist lineages of meristematic cells and *STM* expression is an essential hallmark of the meristematic cell lineages.

AM formation involves multiple transcription factors and is finely tuned and shaped by a variety of phytohormones including auxin and cytokinin. Besides these biochemical signals, it takes place in the crease-like boundary region and is exposed to and regulated by strong anisotropic mechanical stress-derived signals. Here I review the recent research proceedings about meristematic cell lineage, which expresses *STM*, mainly from the perspectives of phytohormones, transcription factors, and mechanical signals arising from the boundaries. However, due to the limit of space, I may not be able to cover all the relevant literature, and further refer readers to excellent review articles that elaborate more on SAM stem cell maintenance, and/or boundary formation (Ha et al., 2010; Hepworth and Pautot, 2015; Wang et al., 2016; Pfeiffer et al., 2017).

## The Roles of Phytohormones

Phytohormones are key regulators in plant growth and development. Phytohormones that play prominent roles in AM initiation include auxin, cytokinin (CK), gibberellins (GA) and possibly brassinosteroids (BR).

Auxin is essential in plant development, especially in organ initiation and growth. On the other hand, the absence or low levels of auxin is also necessary for shoot meristem maintenance, axillary meristematic cell competence maintenance, as well as subsequent axillary meristem initiation. PIN protein-mediated auxin transport is essential in creating such minima (Wang Q. et al., 2014; Wang Y. et al., 2014). However, auxin signaling is also required for stem cell activation, i.e., the second step in AM initiation. A recent study in *Arabidopsis* found that AM initiation is severely compromised in a dominant gain-of-function allele of *MONOPTEROS* (*MP*), encoding a class A Auxin Response Factor (Guo et al., 2020). On the one hand, AM initiation is also mildly reduced in a hypomorphic loss-of-function *mp* allele. Whereas *STM* is no longer maintained in the gain-of-function *MP*Δ allele, *STM* expression maintenance is largely unaffected in the loss-of-function allele, suggesting that auxin is required for the second step of AM initiation (Guo et al., 2020). MP may activate the expression of *PINHEAD/ARGONAUTE10*, whose protein product sequesters miR165/166, and can promote AM initiation (Mcconnell and Barton, 1995; Lynn et al., 1999; Zhang C. et al., 2020). Nevertheless, it needs to be tested if the MP activation activity functions in the leaf axil or elsewhere. Consistent with the roles of auxin in promoting the second step of AM initiation, maize auxin biosynthesis mutants are defective in vegetative AM initiation (Matthes et al., 2019). Together, an initial auxin minimum followed by a low level of auxin signaling is required for AM initiation. It should be noted that multifaceted functions of auxin are not only required in vegetative AM initiation, but also in SAM homeostasis, evidenced by the low auxin levels and signaling outputs in the central zone and that the ectopic expression of the dominant auxin-insensitive form of an AUX/IAA repressor, BODENLOS, in the stem cell niche causes SAM termination (Ma et al., 2019).

CK has been implicated in many developmental processes involving cell proliferation and differentiation. CK regulates AM initiation and particularly plays a role in the activation of stem cells required for AM function. Similar to its essential presence in SAM (Shi and Vernoux, 2021; Yang et al., 2021), its high signaling strength is also required in AM (Wang Y. et al., 2014; Wang et al., 2017). It remains to be determined for AMs whether CK promotes cell proliferation through nuclear shuttling of Myb-domain protein 3R4 (MYB3R4) as in SAM (Yang et al., 2021). Consistently, mutations in type-B ARRs result in defective AM formation, probably due to the failure in activating WUS expression (Wang Y. et al., 2014; Wang et al., 2017).

A recent study suggested a critical role of a third phytohormone, GA, in repressing axillary meristem formation through the action of DELLA proteins and miR156/SQUAMOSA-PROMOTER BINDING PROTEIN LIKE 9 (SPL9) module (Zhang Q. Q. et al., 2020). The crosstalk and balance between GA metabolism and LAS precisely regulate AM formation at spatial and temporal resolutions. Low GA levels in leaf axils are crucial to maintain the AM initiation ability. It remains to be answered whether GA counteracts with CK in stem cell competence maintenance or activation. AM initiation deficiency of GA mutants are relatively weak, implying indirect regulations or genetic redundancy.

The fourth phytohormone that may regulate AM initiation is BR, which promotes cell enlargement and differentiation, and is required for organ morphogenesis (Clouse and Sasse, 1998). BR-activated transcription factor BRASSINAZOLE-RESISTANT1 (BZR1) represses the expression of the boundary specific genes such as *CUP-SHAPED COTYLEDON* (*CUC*) and *LATERAL ORGAN FUSION 1* (*LOF1*) genes (Lee et al., 2009; Gendron et al., 2012) that promote AM initiation, and therefore may mediate BR-dependent suppression of stem cell fate. PHYB ACTIVATION TAGGED SUPPRESSOR1 (BAS1), a BR-inactivating enzyme, is specifically activated in organ boundaries and leads to reduced BR response (Bell et al., 2012), which, combined with the absence of BZR1 in boundaries, suggests that BR plays a positive role in organ specification and a negative role in meristematic cell fate maintenance. However, BZR1 is also expressed in inflorescent SAM, suggesting that BR may work in a more complex fashion to repress stem cell lineage in boundaries, or that during reproductive stages BR play roles other than repressing SAM indeterminacy. In fact, AM deficiency is only marginal in BR mutants. As such, the roles of BR in AM initiation warrant further clarification.

## Gene Regulatory Network Composed of Transcription Factors and Epigenetic Regulators

Genetic studies in *Arabidopsis thaliana*, tomato (*Solanum lycopersicum*), rice (*Oryza sativa*), and maize (*Zea mays*) revealed a handful of transcription factors involved in axillary meristem initiation. These transcription factors include CUC, LATERAL SUPPRESSOR (LAS), REGULATOR OF AXILLARY MERISTEMS (RAX), REVOLUTA (REV), and REGULATOR OF AILLARY MERISTEM FORMATION (ROX) in Arabidopsis and tomato, and their orthologs in monocots (Rick and Butler, 1956; Williams, 1960; Talbert et al., 1995; Greb et al., 2003; Li et al., 2003; Keller et al., 2006; Muller et al., 2006; Raman et al., 2008; Yang et al., 2012). They comprise important gene regulatory networks (GRNs) for AM initiation. Cell type-specific TRAP-seq studies revealed LAS and CUC as the dual hubs of the AM-regulatory GRNs (Tian et al., 2014), the expansion of which still awaits more network components to emerge. The genetic interactions between these factors and other executive factors ensure relatively robust AM initiation. Malfunction in these transcription factors results in defective AM formation, much of which is associated with failure in meristematic cell maintenance or stem cell activation (Shi et al., 2016; Wang et al., 2017). For example, in *las* mutants, *STM* mRNA is diminished from the middle regions in boundaries, where AMs are supposed to arise from Greb et al. (2003). Its accumulation in early stages is not affected, suggesting that the up-regulation of *STM* expression and subsequent stem cell niche re-establishment may be affected.

**FIGURE 1:**
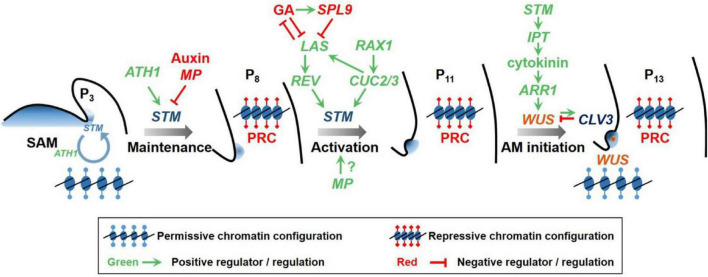
A conceptual model of the two-step regulation of AM initiation. Low levels of *STM* expression is maintained in early leaf primordium (P_3_) axils to reserve the competence of leaf axil cells to form AMs. High levels of auxin as well as unleashed auxin signaling (represented by *MP*) suppress *STM* expression and cell competence. In more mature leaf primordia (P_8_), the expression of *REV*, which is regulated by *LAS*, up-regulates *STM* expression to promote AM initiation. GA suppresses *LAS* expression through the action of SPL9, and *vice versa*, LAS suppresses GA biosynthesis. In parallel, *RAX1* promotes *CUC2/3* expression, which in turn activates *STM* and *LAS* expression. STM promotes cytokinin biogenesis, which, during the AM initiation stage, then activates WUS expression *de novo* through type B-ARRs to enable stem cell specification and axillary bud formation. WUS activates the expression of the secreted peptide *CLV3*, which in turn downregulates *WUS* expression through the Leucine-rich repeat receptor kinase CLV1. Epigenetic modification is involved in restricting gene expression in the leaf axil. In each part of the diagram, the red inhibition symbols indicate transcriptional repression, and the green arrows indicate transcriptional activation. Modified from Wang and Jiao (2018).

Intriguingly, all the transcription factors seem to converge onto the same gene regulator, *STM*, to regulate AM initiation. The basal level of *STM* expression is necessary to maintain the morphogenetic competence in leaf axils (Burian et al., 2016; Shi et al., 2016). At this stage, STM protein and its interacting ARABIDOPSIS THALIANA HOMEOBOX GENE1 (ATH1) protein form a heterodimer, which binds and keeps permissive chromatic environment at the *STM* locus, thus forming a self-activation loop (Cao et al., 2020). Such an auto-regulation loop allows the rapid activation of *STM* transcription in the following phase. The elevation in *STM* expression levels further requires the HD-ZIP III family transcription factor REV in the second step of AM initiation. Because STM promotes CK biosynthesis (Jasinski et al., 2005; Yanai et al., 2005; Coudert et al., 2019), elevated levels of STM are required for a CK signaling maxima in AM, which in turn is required to activate *WUS* expression (Figure 1; Wang Y. et al., 2014; Wang et al., 2017). Together, these genes mark the establishment of a new functional SAM. Once established, the stem cell niche of AM may be self-maintained through a plasmodesmata-dependent non-cell-autonomous WUS transport, as in SAM (Yadav et al., 2011; Daum et al., 2014). In addition, we also expect that CLV3-mediated WUS nuclear-cytoplasmic partitioning also regulates WUS gradient in the AM organization center and in turn affects its own expression and the stem cell homeostasis as in SAM (Plong et al., 2021). The positive feedback between WUS and CK through type-A ARRs (Leibfried et al., 2005) also remains to be tested for AMs.

The differential *STM* and *WUS* expression timing patterns are achieved through epigenetic modifications around the promoter region. In *Arabidopsis*, Polycomb Group Repressive Complex 1 (PRC1) and PRC2 are actively involved in *STM* locus modification in mature leaf cells and mediate transcriptional repression (Figure 1; Shi et al., 2016). Similarly, they also establish the repressive environment around *WUS* gene locus (Figure 1; Wang et al., 2017). By contrast, in tomato, PRC2 component Super Determinant 1A (Sde1A) and PRC1 component B cell-specific Moloney murine leukemia virus integration site 1 (Bmi1) collaborate synergistically in promoting but not suppressing AM formation, through impacting associated histone marks (Lopez et al., 2021). This finding also implies a complicated constitution and diverged roles of PRC1 and PRC2 complexes.

## The Roles of Boundaries

The meristem-leaf boundaries from which axillary meristems arise are essential in separating fully differentiated organs or differentiating primordia and SAM, and therefore are important for stem cell activity and plant stature patterning. Boundary zones are saddle-shaped and tightly packed with cells with restricted division and growth (Hussey, 1971b; Reddy et al., 2004). The special curvature and specific gene expression patterns of boundary zones are important for its function as separation between SAM and peripheral organs (Hepworth and Pautot, 2015; Wang et al., 2016).

Intriguingly, the transcription factors and hormonal signaling pathways actively functioning in AM (discussed in sections “The Roles of Phytohormones” and “Gene Regulatory Network Composed of Transcription Factors and Epigenetic Regulators”) are also actively modulated in the boundaries. Consistent with their consequential occurrence, in mutants defective in boundary formation, AM initiation may also be disrupted, suggesting that these two developmental processes are often regulated by shared factors. For instance, in *las* loss-of-function mutants, the fusion between leaf petiole and stem is observed and no AMs form in most leaf axils (Greb et al., 2003). Similar defects were observed in *cuc* mutants (Raman et al., 2008). The fusion between leaves and stem is constituted by over-proliferating cells. AM initiation is also mostly abolished in *cuc* mutants (Raman et al., 2008). Nevertheless, it needs to be noted that while some boundary mutants fail in organ separation and AM initiation simultaneously, others do not necessarily fail in AM initiation, as observed in mutants for *Lateral Organ Boundaries* (*LOB*) and its closely related *Jagged Lateral Organs* (*JLO*) (Shuai et al., 2002; Borghi et al., 2007). *JLO* controls organ patterning and separation through modulating auxin distribution and signaling (Borghi et al., 2007; Bureau et al., 2010; Rast and Simon, 2012).

**FIGURE 2:**
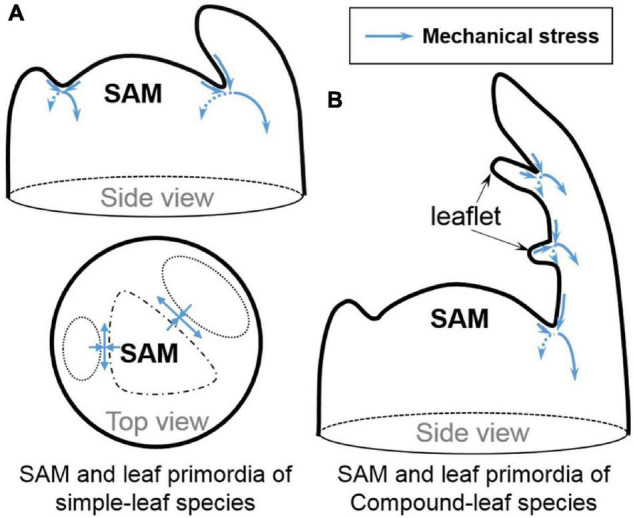
Stress patterns in boundary regions. **(A)** The anisotropic stress (blue arrows) in the boundary regions between primordia and SAM. **(B)** The possible anisotropic stress (blue arrows) between leaftlets in compound-leaf species. Modified and inferred from Hussey (1971a); Hamant et al. (2008) and Sampathkumar et al. (2014).

Auxin counteracts with boundary-promoting genes in boundary formation. When auxin synthesis is ectopically induced in leaf axils driven by the *LAS* or *CUC* promoters, boundaries are significantly widened and boundary cell morphology has greatly changed, connecting cell fate shift and boundary geometry reshaping, both resulting from auxin induction (Wang Q. et al., 2014). Besides the GRNs comprising transcription factors and phytohormones, mechanical signals may affect cell fate. Stem cell re-activation also seems to be regulated directly by mechanical signals conferred by the boundary curvature independent of the auxin minimum. The saddle-shaped boundary curvature provides an important mechanical signal (Figure 2A; Sampathkumar et al., 2014) that can be uncoupled from auxin depletion from the boundary domain to induce strong *STM* expression (Landrein et al., 2015; Landrein and Ingram, 2019). Furthermore, *CUC3* expression in boundaries is also induced by mechanical stress (Fal et al., 2016). It remains to be tested whether other biochemical signals are regulated in the same way and whether mechanical signals change the chromatin accessibility and the epigenetic marks thereof.

Boundary domain establishment not only affects AM formation, it is also recruited in compound leaf development in species such as tomato, another example of fractal pattern. In tomato compound leaves, boundaries between leaflets are characterized by suppressed cell division and reduced cell growth (Hussey, 1971b; Reddy et al., 2004) and shaped by multiple AM regulators including, *Lateral Suppressor* (*LAS* ortholog), *Goblet* (*CUC* ortholog), *Blind*/Potato Leaf (*C*) (RAX ortholog), auxin and cytokinin (Brand et al., 2007; Berger et al., 2009; Busch et al., 2011). Ectopic meristems can form at the leaflet boundaries (Rossmann et al., 2015), suggesting that these boundaries share similar molecular and possibly mechanical characteristic as leaf-SAM boundaries (Figure 2B) and that cells at leaflet boundaries remain meristematic. It is tempting to suggest that there might be a universal mechanism governing meristematic cell maintenance or activation across these distinct boundaries. It would be interesting to test if external forces generated through micromechanical perturbations could also induce *STM* expression and even ectopic meristem formation between leaflets.

## Perspectives

In summary, recent breakthroughs in the field of AM initiation have highlighted the importance of meristematic cell lineage maintenance in branching, and establish AM initiation as an excellent system to study cell lineage, which has broad applications in stem cell biology. There are still many open questions to be addressed before the molecular and mechanical signals can be united to answer the question of indeterminacy maintenance and activation.

It is obvious that many regulators in SAM development also regulate AM formation, with a few awaiting further verification and characterization. For example, the state of nutrients such as nitrate and phosphorus, patterns stem cell dynamics and SAM activity (Landrein et al., 2018). As plant growth environment greatly changes plant architecture, it certainly could do so through affecting AM formation and activity. It remains to be investigated whether nutritious state also affects AM formation. Likewise, whether the highly conserved energy-sensing TARGET OF RAPAMYCIN (TOR) pathway, which is recruited to integrate light and metabolic signals for SAM stem cell activation (Pfeiffer et al., 2017), is also deployed in stem cell activation for AM initiation remains an open question.

Furthermore, given the incompleteness of GRNs governing meristematic cell lineage maintenance and activation, more genetic components are likely to emerge with the aid of single-cell RNA-seq, which helps to construct GRNs at the cellular-resolution. Forward and reverse genetic screenings are also necessary and critical to identify more genetic components, which have long been missing in the model plant *Arabidopsis*.

## Author Contributions

YW wrote and edited the manuscript.

## Conflict of Interest

The author declares that the research was conducted in the absence of any commercial or financial relationships that could be construed as a potential conflict of interest.

## Publisher’s Note

All claims expressed in this article are solely those of the authors and do not necessarily represent those of their affiliated organizations, or those of the publisher, the editors and the reviewers. Any product that may be evaluated in this article, or claim that may be made by its manufacturer, is not guaranteed or endorsed by the publisher.
